# Energy Availability and Alcohol-Related Liver Pathology

**Published:** 2003

**Authors:** Carol C. Cunningham, Cynthia G. Van horn

**Affiliations:** Carol C. Cunningham, Ph.D., is professor of biochemistry, and Cynthia G. Van Horn, Ph.D., is a postdoctoral fellow, both in the Department of Biochemistry at Wake Forest University School of Medicine, Winston-Salem, North Carolina

**Keywords:** chronic AODE (alcohol and other drug effects), alcoholic liver disorder, oxygen, bioavailability, energy, liver, hepatocyte, ATP (adenosine triphosphate), metabolism, mitochondria, glycolysis, oxidative phosphorylation, pathogenesis

## Abstract

Alcohol consumption alters the metabolism of the most common type of cell found in the liver, the hepatocyte. The presence of alcohol in the body causes the liver to use more oxygen—for example, when breaking down the alcohol. Increased oxygen use, in turn, causes oxygen deficits in several key cells, particularly in hepatocytes located near the small hepatic veins. These veins return blood to the heart for re-oxygenation after it has passed through the liver. Hepatocytes surrounding these veins are the first to show signs of liver disease. The damage induced by oxygen deficits may be exacerbated by alcohol-induced deficits in other components that are essential for cell survival. For example, adenosine triphosphate (ATP), the cell’s main source of energy, is generated primarily during the course of two sets of metabolic reactions: glycolysis and the mitochondrial oxidative phosphorylation process. Alcohol consumption may interfere with both of these pathways of ATP production through several mechanisms. An inadequate supply of ATP impairs the cell’s ability to perform critical functions, including the repair of alcohol-induced cell damage, and may therefore contribute to cell death and alcoholic liver disease.

A substantial amount of evidence indicates that alcoholic liver disease develops when alcohol alters the cellular environment of the liver, thereby initiating abnormal interactions among various types of liver cells. According to one prominent hypothesis, alcohol causes changes to the walls of the intestine, which allows a harmful bacterial product called endotoxin to pass into the blood more readily (Tsukamoto and Kaplowitz 1996). As a result, endotoxin levels in the blood and tissues rise. The body responds to this increase in endotoxin by launching a coordinated immune response. For example, high endotoxin levels in the liver cause immune cells residing in the liver (Kupffer cells) to release signaling molecules (i.e., cytokines) as well as other compounds (e.g., prostaglandins) that result in a stepped-up inflammatory response. (For more information on endotoxin and its effects on Kupffer cells, see the article in this issue by Wheeler.) Cytokines and prostaglandins, in turn, increase the metabolic activities of liver cells, especially the hepatocytes, which account for approximately 90 percent of the liver cell mass. When their metabolism increases, the cells require more oxygen and fuel (nutrients) to keep pace with this increased metabolic demand. Oxygen is required for many biochemical reactions in the cell, and the breakdown of nutrients provides the energy needed for these reactions. In addition, the breakdown of alcohol itself, which occurs primarily in the hepatocytes, increases the liver’s need for oxygen, as described in the next section.

Under normal circumstances the blood supplies enough oxygen to the liver, but if hepatocytes use up more oxygen because of the breakdown of alcohol, oxygen deficits (i.e., hypoxia) can develop in some liver areas. Hypoxia, in turn, may impede the liver cells’ ability to produce an energy-rich molecule called adenosine triphosphate (ATP), which is generated during the breakdown of nutrients and supplies energy needed for numerous biochemical reactions. Sufficiently high levels of ATP are essential to the survival of all cells; reduced ATP levels in the liver are one factor contributing to liver cell death and may contribute to development of alcoholic cirrhosis.

This article describes alcohol’s effects on hepatocyte metabolism and oxygen use, reviewing the consequences of alcohol-related hypoxia on ATP levels in the liver and summarizing alcohol’s specific effects on the two main cellular pathways of ATP production.

## Effects of Alcohol Consumption on Oxygen Use in the Liver

Alcohol consumption can increase the liver cell’s use of oxygen both indirectly and directly. The indirect pathway is associated with the alcohol-induced activation of immune cells (Kupffer cells) that reside in the liver. When Kupffer cells become activated, they release various signaling and stimulatory molecules, including prostaglandin E2. This molecule can stimulate the metabolic activity of the hepatocytes. This metabolic activity consists of breaking down and synthesizing many essential molecules and cell components, and the chemical reactions involved in these processes frequently involve oxygen molecules (i.e., are oxidation and reduction reactions). (For more information on these reactions, see the sidebar “Oxidation and Reduction Reactions.”) Thus, more active metabolism in the liver increases the need for oxygen. Animal studies have yielded results consistent with this scenario, showing that oxygen use in the liver increases after both acute and chronic alcohol administration (Yuki and Thurman 1980; Arteel et al. 1996; Videla et al. 1973).

**Oxidation and Reduction Reactions**

The breakdown of nutrients such as carbohydrates, proteins, and fats, as well as other molecules such as alcohol, frequently involves chemical reactions that use oxygen and/or hydrogen (i.e., oxidation and reduction reactions). Generally speaking, oxidation reactions are those in which an oxygen atom is added to a molecule, hydrogen atoms are removed from a molecule, or electrons are removed from a molecule. (Electrons are the negatively charged particles in each atom.) In reduction reactions, the reverse occurs—that is, an oxygen atom is removed or hydrogen atoms or electrons are added. Oxidation and reduction reactions always occur together: When, for example, electrons or hydrogen atoms are removed from molecule A and transferred to molecule B, molecule A has been oxidized, whereas molecule B has been reduced.

**Figure 1:**
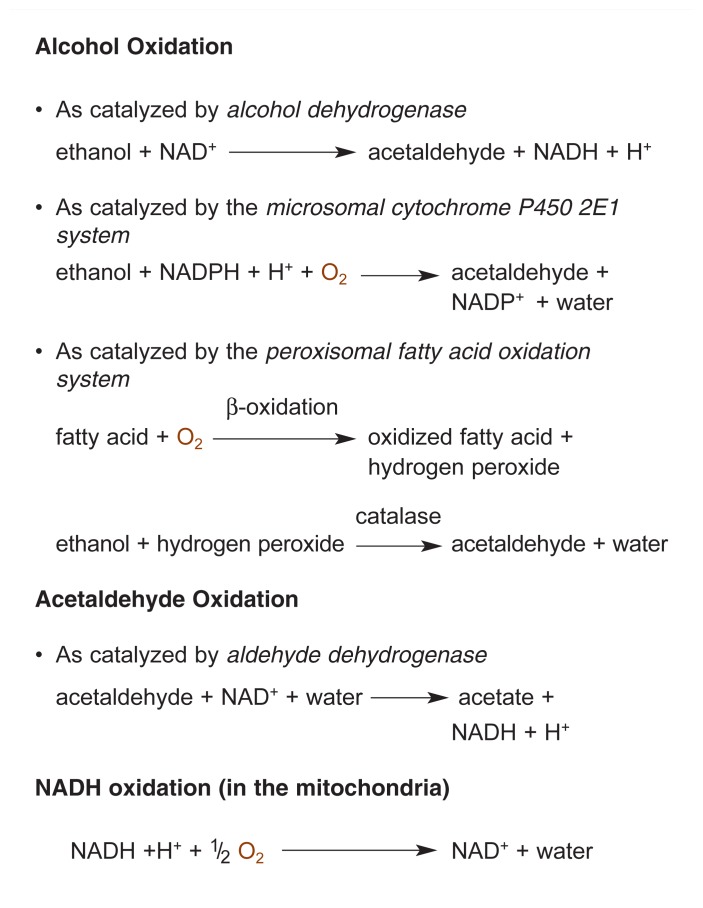
Oxygen utilization associated with various pathways of alcohol (ethanol) oxidation in the liver. NOTE: NAD = nicotinamide adenine dinucleotide; NADH = reduced NAD; NADP = nicotinamide adenine dinucleotide phosphate; NADPH = reduced NADP.

Alcohol (chemically known as ethanol) is metabolized by several different reactions in the liver, most of which involve oxidation/reduction reactions (see figure 1 in the main article). The predominant process involves two oxidation reactions. First, the enzyme alcohol dehydrogenase converts alcohol to acetaldehyde by removing two electrons. Then, another enzyme, aldehyde dehydrogenase, converts the acetaldehyde into acetate by removing two additional electrons, after which a hydroxyl ion from a water molecule is added (see figure 1). The electrons that are removed during these reactions are transferred to a molecule called nicotinamide adenine dinucleotide (NAD), which is thereby converted to reduced NAD (NADH). NADH can then participate in other metabolic reactions, including reduction of oxygen to H_2_O, which is accomplished by the respiratory chain in the mitochondria. During these reactions, NADH releases its electrons (another oxidation reaction), becoming available again as an electron acceptor (see figure 1).

— *Carol C. Cunningham and Cynthia G. Van Horn*

In addition to these indirect effects, alcohol directly enhances the liver’s oxygen use through its own breakdown in the hepatocytes. Alcohol (chemically known as ethanol) can be broken down with the help of several enzyme systems, including alcohol dehydrogenase, the cytochrome P450 system, and the fatty acid–catalase system (Cunningham and Bailey 2001).^1^ Each of these systems oxidizes alcohol—that is, the chemical reaction involved uses oxygen (O_2_), or removes electrons from the alcohol molecule or its degradation products, or both (see figure 1). Of these three systems, the alcohol dehydrogenase system breaks down most of the alcohol, particularly after moderate alcohol use.

Alcohol dehydrogenase, together with another enzyme, aldehyde dehydrogenase, breaks down alcohol to form acetate and water. During this process, electrons are transferred to a compound called nicotinamide adenine dinucleotide (NAD) to generate reduced NAD (i.e., NADH) (see figure 1; for more information see the sidebar “Oxidation and Reduction Reactions”). NADH, in turn, can transfer the newly acquired electrons to the first of a series of electron transport components composing the respiratory chain, which is found in a cell structure called the mitochondrion (Berg et al. 2002). The electrons are eventually transferred to O_2_, which then binds protons (H^+^) to generate water (H_2_O). Therefore, cells (i.e., hepatocytes) in which alcohol is oxidized need additional oxygen, as illustrated in figure 1. (For more information on the respiratory chain, see the sidebar “Pathways of ATP Production” and the section “Alcohol-Induced Damage to Liver Mitochondria.”)

**Pathways of ATP Production**

As mentioned in the main article, a molecule called adenosine triphosphate (ATP) is the primary source of the energy that is needed for numerous biochemical reactions in the cell. ATP is formed when nutrients are broken down, a process that releases energy which then can be used to produce ATP. One nutrient that is a primary source of ATP production is the sugar glucose, which either can be imported into the cell from the blood or generated by the breakdown of glycogen, a large molecule that serves as a means of storing glucose in the cells. Glucose degradation occurs in several steps that take place in different areas of the cell (see the figure). In an initial series of reactions, referred to collectively as glycolysis, glucose is converted to a molecule called pyruvate. This process, which occurs in the fluid filling the cell (i.e., the cytosol), provides enough energy to generate a relatively small amount of ATP (i.e., two molecules of ATP for each glucose molecule broken down to pyruvate).

The subsequent fate of the pyruvate depends on the oxygen conditions in the cell. When oxygen levels are low, pyruvate is converted to lactate, which can accumulate in the cell; this reaction generates no further ATP. If enough oxygen is present, however, pyruvate is transported into mitochondria— small membrane-enclosed cell structures that act as the cell’s power plants. In the mitochondria, pyruvate enters a chain of biochemical reactions collectively called the citric acid cycle, which ultimately degrade pyruvate to carbon dioxide (CO_2_). During these reactions (which are called oxidation reactions; see the sidebar on oxidation and reduction) electrons are released from intermediate reaction products and transferred to a molecule called nicotinamide adenine dinucleotide (NAD) to yield reduced NAD (NADH).

**Figure:**
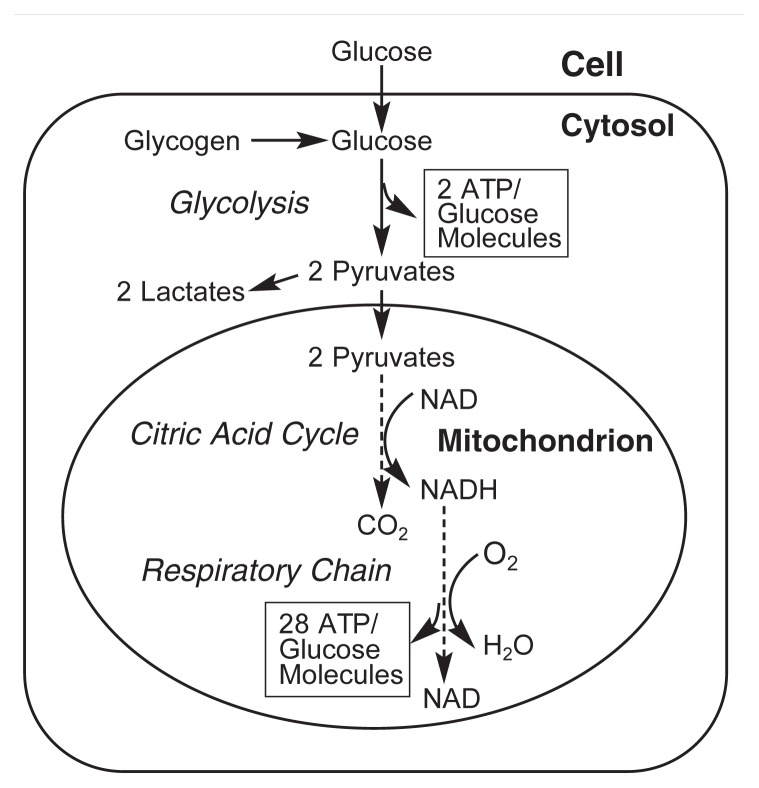
Each step in the process of metabolizing glucose and generating ATP involves several biochemical reactions.

NADH can then feed the electrons into the mitochondrial electron transport system (also called the respiratory chain). In this electron transport system, the electrons are released from the NADH and transferred to a series of other molecules that first accept the electrons and then pass them on to the next molecule in the chain. Finally, the electrons (together with protons [H^+^]) are transferred to oxygen to generate water. These successive electron transfer reactions provide enough energy to drive the formation of additional ATP molecules. In fact, the mitochondrial system generates substantially more ATP than glycolysis, for a total of 28 ATP molecules for every molecule of glucose (or, more specifically, two pyruvates) that enters the citric acid cycle. The net result of all of these reactions is the synthesis of ATP from two precursor molecules, adenosine diphosphate (ADP) and inorganic phosphate (Pi). This whole process of ATP production, which is illustrated in the accompanying figure, requires numerous oxidation and reduction reactions combined with the reaction of Pi with ADP, and is known as oxidative phosphorylation.

— *Carol C. Cunningham and Cynthia G. Van Horn*

Together, alcohol breakdown in the hepatocytes and the indirect effects of alcohol that are mediated by Kupffer cells cause the liver to use more oxygen than normal during alcohol consumption. Other mechanisms by which alcohol influences the liver’s oxygen use probably also exist, but for the processes discussed in this section, more detailed experimental evidence has been obtained to date.

**Figure 2:**
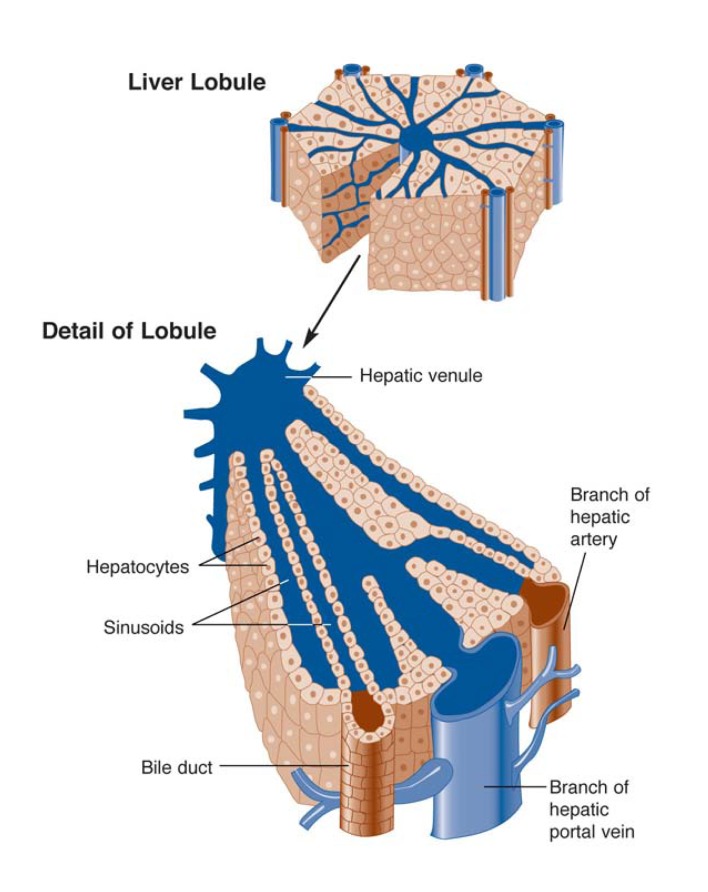
The structure of the liver’s functional units, or lobules. Blood enters the lobules through branches of the portal vein and hepatic artery, then flows through small channels called sinusoids that are lined with primary liver cells (i.e., hepatocytes). The hepatocytes remove toxic substances, including alcohol, from the blood, which then exits the lobule through the central vein (i.e., the hepatic venule). SOURCE: Adapted from Ross et al. 1995.

**Figure 3:**
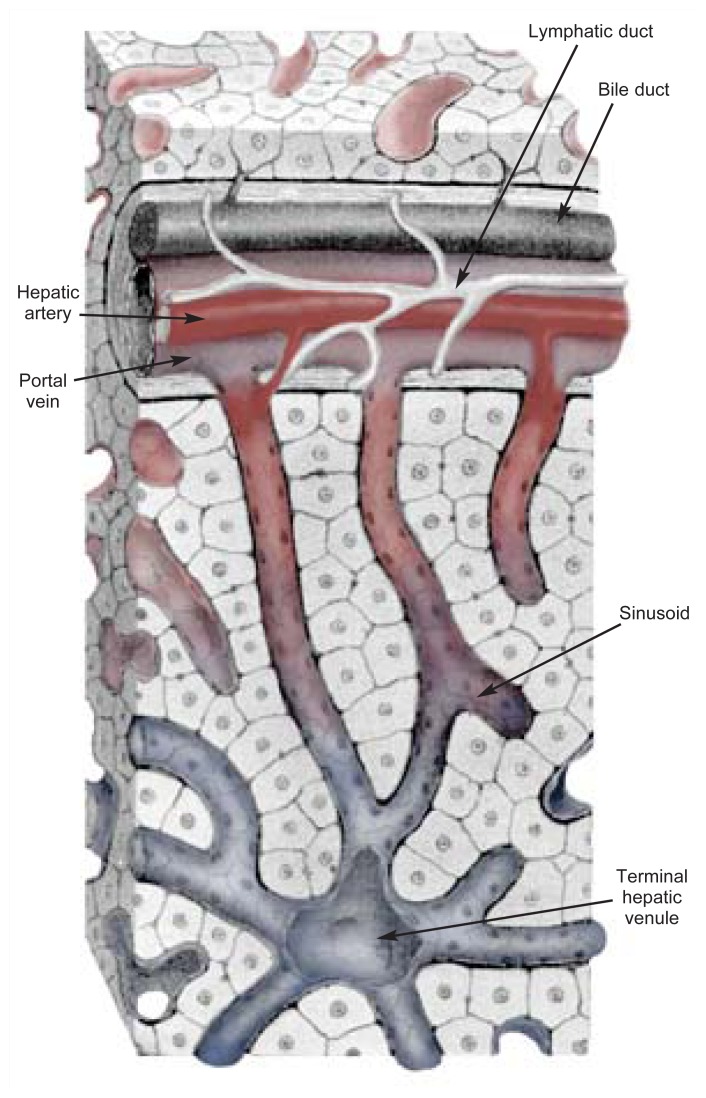
The liver lobule in more detail. The blood entering the lobule (at the hepatic artery, indicated in red) is relatively oxygen rich, but the blood leaving the lobule contains only low levels of oxygen (at the terminal hepatic venule, indicated in blue) because hepatocytes along the sinusoids have used up much of the available oxygen. SOURCE: Adapted from Rubin and Farber 1994, with permission. Artist: Dimitri Karetnikov.

The alcohol-related increase in the liver’s use of oxygen exacerbates the normal differences in oxygen levels found within each of the basic structures, or lobules, of the liver (Junqueira et al. 1998). The entire liver consists of many thousands of these lobules, each of which is made up of hundreds of hepatocytes and other types of cells (see figure 2). Each lobule exhibits the same pattern of blood flow: Nutrient- and oxygen-rich blood enters the liver from the portal vein and hepatic artery and is distributed to all the lobules. Within each lobule, the blood flows past the hepatocytes through small channels called sinusoids before exiting the lobule through the hepatic venule, a small vein located in the center of each lobule. As a result, hepatocytes located near where the blood enters the lobule—that is, close to the portal vein and hepatic artery (i.e., the periportal hepatocytes)—are exposed to the most nutrient- and oxygen-rich blood. Liver cells closer to where the blood exits the lobule at the hepatic venule (i.e., perivenous hepatocytes) are exposed to blood containing less oxygen because much of the oxygen and nutrients already has been extracted from the blood by other hepatocytes. Thus, even under normal metabolic conditions— that is, in the absence of alcohol—oxygen levels vary in different regions of the liver lobule, with high concentrations in the periportal cells and lower levels in the perivenous hepatocytes (see figure 3).

When alcohol is consumed and subsequently broken down in the liver, the oxidation reactions involved lead to even lower concentrations than normal in the perivenous region of the lobule (Sato et al. 1983; Arteel et al. 1996). The same region also is the first to show liver cell death after chronic alcohol consumption (Ishak et al. 1991), suggesting that an oxygen deficit in this region may be a risk factor for the development of alcoholic liver disease.

One possible consequence of this oxygen deficit may be a decrease in the capacity of the perivenous hepatocytes to produce normal ATP levels that are necessary to the cells’ ability to survive. Researchers have not yet analyzed ATP levels specifically in the perivenous cells of alcohol consumers; however, they have evaluated the effect of chronic alcohol consumption on ATP concentrations in the liver as a whole, as described in the following section.

## Alcohol Abuse Reduces Liver ATP Concentrations

All cells require energy to fulfill their diverse functions and to ensure the viability of the cells themselves and the entire organism. This energy is derived from the metabolism of nutrients, such as carbohydrates, proteins, and fats. When these nutrients are broken down, energy is released that is used to make ATP, which in turn can provide the energy to other reactions. Some forms of the nutrients can be stored in the cells so that they can be broken down to generate ATP whenever energy is needed for cellular reactions. The ATP itself cannot be stored in the cells; nevertheless, measuring ATP levels in a tissue provides researchers with a “snapshot” of the tissue’s metabolic activity and health.

Oxygen levels in the tissue (i.e., the oxygen environment) can influence the concentrations of ATP in the liver, particularly in animals that chronically consume alcohol. For example, researchers evaluated ATP levels in livers from rats maintained on an alcohol-containing diet for 1 month and in livers from control animals that did not receive alcohol. These studies found that when oxygen levels in isolated hepatocytes or larger sections of the liver were normal, both groups of animals also had normal ATP levels in their livers. When the cells or tissues had oxygen deficits, however, ATP levels in the liver were reduced much more dramatically in the alcohol-consuming than in the control animals (Spach et al. 1991; Bailey and Cunningham 1999). Other investigators made similar observations with living animals that had received high doses of alcohol for extended periods of time. After a period of oxygen deficiency, the livers of alcohol-treated animals showed greater reductions in ATP levels than did those of control animals (Miyamoto and French 1988). These findings indicate that chronic alcohol consumption increases the sensitivity of liver cells to oxygen deficits, resulting in decreased ATP concentrations in the cells. The results also suggest that ATP concentrations may decrease in any oxygen-deficient region of the liver in chronic drinkers, such as the perivenous region of each lobule.

An alcohol-related reduction in ATP concentrations in the perivenous region might predispose this area of the lobule to scar tissue formation (i.e., fibrosis) as well as to cell and tissue death (Ishak et al. 1991). The effects of reduced ATP levels are particularly dramatic in the livers of alcohol users because ATP is needed to provide energy for the repair of cell structures and complex molecules that have been damaged by alcohol or its breakdown products. Alcohol metabolism gives rise to numerous compounds that are toxic to the cells, including the following:

- Acetaldehyde, which can interact with proteins and other complex molecules. (For information on the role of acetaldehyde and other reactive molecules generated during alcohol metabolism, see the article by Tuma and Casey in this issue.)
- Highly reactive, oxygen-containing molecules collectively called reactive oxygen species (ROS), such as the hydroxyl radical, superoxide, and hydrogen peroxide. (For more information on the role of ROS in liver damage, see the article by Wu and Cederbaum in this issue.)
- Other highly reactive molecules, such as the hydroxyethyl radical, peroxynitrite, and lipid peroxides.

All of these diverse compounds react with and damage complex molecules in the cells—such as proteins, phospholipids (which are central components of the cell membrane), and nucleic acids (e.g., DNA). The damaged molecules then must be replaced or repaired to ensure the cell’s survival. These repair processes all require energy. Consequently, the cells’ demands for ATP increase even further following chronic alcohol consumption.

Two biochemical pathways in the cell account for all ATP synthesis. Under normal conditions, most of the ATP in the liver is generated by a chain of reactions known as the mitochondrial oxidative phosphorylation system, which is composed of the respiratory chain and other enzymes. The remaining ATP is produced by the glycolysis pathway, which consists of a series of chemical reactions involved in the breakdown of the sugar glucose (Berg et al. 2002). (For more information on these two pathways, see the sidebar “Pathways of ATP Production.”) It is well established that chronic alcohol use adversely affects both the structure and the function of the mitochondria in liver cells, thereby interfering with the oxidative phosphorylation system. Other evidence indicates that alcohol also interferes with glycolysis, resulting in reduced ATP synthesis, particularly in the presence of oxygen deficits. Both of these consequences of chronic alcohol use are discussed in the following sections.

### Alcohol-Induced Damage to Liver Mitochondria

Most of the cell’s supply of ATP is generated in mitochondria, which therefore are considered the cell’s power plants. Researchers found that changes in the structure and function of hepatic mitochondria are early consequences of chronic alcohol consumption (Cunningham et al. 1990; Hoek 1994). For example, the mitochondria can swell to an abnormal size following chronic alcohol consumption. Alcohol also can change the composition of the phospholipids that make up the mitochondrial membranes, although it is not known whether these changes influence mitochondrial functioning. Finally, alcohol can alter the protein content of the mitochondria, and these alterations interfere with the mitochondria’s ability to synthesize ATP.

The respiratory chain consists of a series of proteins that can transfer negatively charged particles (i.e., electrons) or hydrogen from energy-rich, oxidizable compounds (e.g., pyruvate generated by the breakdown of the sugar glucose, and fatty acids generated by the breakdown of dietary fats) to O_2_ (Berg et al. 2002). This process releases energy that can be used to generate ATP from precursor molecules. Mitochondria from the livers of alcohol-fed animals contain lower amounts of some components of the respiratory chain than do the mitochondria of control animals (Cunningham et al. 1990). In addition, the alcohol-fed animals have lower levels of the enzyme complex that mediates ATP production. As a result, the rate of ATP synthesis in the liver mitochondria decreases as well, leading to an overall decline in ATP concentration in the liver.

When oxygen levels in cells are low, ATP production through the respiratory chain is greatly reduced, and most ATP is produced through glycolysis (as described in the following section). Even under those conditions, however, a sufficient amount of oxygen remains to allow limited mitochondrial synthesis of ATP, at least in healthy cells. When the cells, and particularly the mitochondria, have been damaged by alcohol, however, they may not be able to synthesize ATP at the same rate as normal mitochondria under low-oxygen conditions. To investigate this possibility, researchers currently are studying whether hepatocytes from alcohol-fed animals produce lower ATP amounts in their mitochondria. Preliminary results suggest that when cells from alcohol-consuming animals are maintained under low-oxygen conditions, ATP production by the mitochondria is significantly reduced.

### Effects of Chronic Alcohol Consumption on Glycolysis

Glycolysis is a series of reactions that break down the sugar glucose^2^ into two molecules of a compound called pyruvate. During glycolysis, which is described in more detail in the sidebar “Pathways of ATP Production,” two ATP molecules are generated for each glucose molecule converted to two molecules of pyruvate. The pyruvate then can be broken down further in the mitochondria in a set of reactions collectively called the citric acid cycle. These reactions generate NADH, which, as mentioned earlier, transfers electrons into the respiratory chain, resulting in the release of enough energy to generate additional ATP molecules.

Because the respiratory chain requires oxygen, this pathway of ATP production is less prominent when oxygen concentrations in the tissues are low. Instead, under low-oxygen conditions, the largest proportion of ATP is produced during glycolysis (Berg et al. 2002). This observation is supported by findings that, in the liver, glycolysis occurs primarily in the perivenous region of the lobule, where oxygen levels are lowest, even in the absence of alcohol (Jungermann and Thurman 1992). When oxygen levels in the liver tissues are too low, the immediate breakdown products of glucose, including pyruvate, accumulate in the cells because there is not enough oxygen available to further break down pyruvate via the citric acid cycle (see the sidebar “Pathways of ATP Production”). The accumulating pyruvate subsequently is converted to lactate, which also can accumulate. In fact, the levels of lactate plus pyruvate are considered a measure of glycolytic activity, particularly under hypoxic conditions, but also when the tissues contain sufficient oxygen (Baio et al. 1998; Van Horn and Cunningham 1999).

Researchers have measured the levels of pyruvate and lactate in the livers of alcohol-consuming animals to determine whether chronic alcohol consumption impairs glycolysis. During these analyses, investigators found that the concentrations of lactate plus pyruvate in the hepatocytes of alcohol-fed animals were lower than in cells from control animals, which strongly suggests that chronic alcohol consumption reduces glycolysis (Baio et al. 1998; Van Horn and Cunningham 1999). This effect occurred regardless of whether the cells were analyzed immediately after they were removed from the animals or whether they were first maintained in the presence of either normal or reduced oxygen levels. These observations demonstrate that chronic alcohol consumption impairs glycolysis in hepatocytes both in the presence and absence of oxygen. Moreover, the findings suggest that the alcohol-related deficit in ATP concentrations observed under low-oxygen conditions results not only from alcohol’s effects on the respiratory chain but also from alcohol-related decreases in glycolysis. Preliminary studies in which researchers directly measured ATP concentrations in the cells indicate that hepatocytes of alcohol-consuming animals produce significantly less ATP by glycolysis than do control animals.^3^

One reason why glycolysis is reduced in chronic alcohol users could be that the cells do not contain enough of the starting material, glucose. Glucose can either be imported into the hepatocyte from the blood by specific transport molecules located in the hepatocyte membrane, or it can be generated inside the hepatocyte by breaking down a very large molecule called glycogen, which serves as a storage form of glucose in liver and other cells. Accordingly, reduced glycolysis could be related to lowered cellular glucose uptake and/or glycogen breakdown. The glucose transporter found in the membrane of hepatocytes in the perivenous region is called Glut 1 (Van Horn et al. 2001). This molecule captures glucose in the blood and moves it into the cells, where it is broken down to generate energy or used to produce other compounds such as glycogen (Gumucio et al. 1996).^4^ Researchers have found that the numbers of Glut 1 molecules on the hepatocytes are lower after chronic alcohol consumption (Van Horn et al. 2001). This reduction could limit glucose uptake in perivenous hepatocytes so that less glucose is available for glycolysis.

Reduced levels of glycogen also could lead to reduced glucose availability and glycolysis in liver cells. Various studies of the intact liver (Van Horn et al. 2001), whole liver hepatocytes (Van Horn and Cunningham 1999; Van Horn et al. 2001), and periportal and perivenous hepatocytes (Baio et al. 1998) demonstrated that glycogen levels are greatly reduced in livers of chronic alcohol consumers. Researchers do not yet know the exact reason why glycogen levels are lower after long-term alcohol consumption. Studies have found that, in rats drinking alcohol, substantially less glycogen is synthesized from the available glucose, probably because the enzymes that generate glycogen from glucose are less active in alcohol consumers. In the perivenous hepatocytes, the rate of glycogen synthesis also may be lower because, as previously mentioned, these cells carry fewer molecules of the Glut 1 transporter and therefore can take up less glucose from the blood. (See Van Horn et al. 2001 for more details on the control of liver glycogen concentrations in alcohol consumers.)

The observations discussed in these sections suggest that the perivenous hepatocytes of alcohol consumers experience more severe oxygen deficits, limiting the cells’ ability to generate ATP in the mitochondrial respiratory chain. Therefore, these cells would be particularly dependent on glycolysis to produce the ATP they need. At the same time, however, less glucose is transported into the perivenous hepatocytes, and less glycogen is available to these cells. As a result, glycolysis and the associated ATP generation also would be reduced in the perivenous hepatocytes. Because of these combined mechanisms, the perivenous hepatocytes probably would be the first cells to experience ATP deficits and to show damage resulting from the lack of this essential molecule— a hypothesis that is consistent with the findings obtained when researchers studied liver tissues of alcoholics.

## Summary

Alcohol consumption enhances the liver’s need for oxygen through several mechanisms. For example, alcohol indirectly alters those metabolic events in the liver that are influenced by oxygen levels. These alterations stem at least in part from increases in the communication among cells and are mediated by cytokines and other stimulatory agents that are released by activated Kupffer cells. In addition, the breakdown of alcohol also uses up oxygen and therefore increases the oxygen needs of liver cells. The increased demand for oxygen, in turn, can lead to oxygen deficits at least in certain regions of the liver lobules (i.e., the perivenous region).

Because ATP synthesis, particularly in mitochondria, requires oxygen, oxygen deficits in the liver can lead to reduced ATP production, with potentially detrimental effects to cells. Indeed, studies indicate that ATP synthesis in the liver, both during glycolysis and through the oxidative phosphorylation system, may be reduced when liver tissue or isolated hepatocytes are subjected to low-oxygen conditions. Accordingly, cells in the perivenous region of the liver lobule—which are particularly likely to experience oxygen deficits following alcohol consumption—are predisposed to show alcohol-related decreases in ATP levels. Reduced ATP concentrations, in turn, could limit the cells’ ability to repair damage caused by toxic byproducts of alcohol metabolism.

Taken together, the observations described in this article lead to the following possible scenario: Alcohol breakdown and other effects of alcohol on liver cells increase the cells’ need for oxygen. Higher oxygen consumption by some liver cells leads to oxygen deficits in the environment of other liver cells, particularly perivenous hepatocytes. As a result, these cells cannot maintain ATP levels adequate for normal cell functioning and for the repair of alcohol-induced cell damage. Insufficient amounts of ATP, in turn, predispose the perivenous cells and the entire perivenous region of the lobule to tissue damage and the development of alcoholic liver disease. Although this scenario has yet to be proven experimentally, it is consistent with clinical observations demonstrating that the first signs of alcoholic liver damage appear in the perivenous regions.

## Footnotes

1 Alcohol dehydrogenase is located in the fluid filling the cell (i.e., the cytosol). The cytochrome P450 system is sequestered within tubular structures in the cell called the endoplasmic reticulum, and the fatty acid–catalase system is located in cell structures called peroxisomes.

2 Most of the carbohydrates that are ingested with the diet are first converted to glucose before they undergo final metabolism.

3 Additional investigations have confirmed that the observed effects did indeed result from reduced glycolysis rather than from the fact that the alcohol-containing diets and control diets used in most studies differed in the amounts of carbohydrates (e.g., glucose) they contained (Van Horn and Cunningham 1999).

4 Hepatocytes in the periportal region of the liver lobule, in contrast, actively produce new glucose, which then is exported into the bloodstream primarily by a different glucose transporter molecule called Glut 2.
